# Lifestyle modifications result in alterations in the gut microbiota in obese children

**DOI:** 10.1186/s12866-020-02002-3

**Published:** 2021-01-06

**Authors:** Ky Young Cho

**Affiliations:** Department of Pediatrics, Kangnam Sacred Heart Hospital, Hallym University College of Medicine, Seoul, South Korea

**Keywords:** Obesity, Child, 16S rRNA gene, Gut microbiota, Weight reduction programs

## Abstract

**Background:**

The association between the gut microbiota and pediatric obesity was analyzed in a cross-sectional study. A prospective study of obese children was conducted to assess the gut microbial alterations after a weight change. We collected fecal samples from obese children before and after a 2-month weight reduction program that consisted of individual counseling for nutritional education and physical activity, and we performed 16S rRNA gene amplicon sequencing using an Illumina MiSeq platform.

**Results:**

Thirty-six participants, aged 7 to 18 years, were classified into the fat loss (*n* = 17) and the fat gain (*n* = 19) groups according to the change in total body fat (%) after the intervention. The baseline analysis of the gut microbiota in the preintervention stages showed dysbiotic features of both groups compared with those of normal-weight children. In the fat loss group, significantly decreased proportions of Bacteroidetes phylum, Bacteroidia class, Bacteroidales order, *Bacteroidaceae* family, and *Bacteroides* genus, along with increased proportions of Firmicutes phylum, Clostridia class, and Clostridiales order, were observed after intervention. The microbial richness was significantly reduced, without a change in beta diversity in the fat loss group. The fat gain group showed significantly deceased proportions of Firmicutes phylum, Clostridia class, Clostridiales order, *Lachnospiraceae* family, and *Eubacterium hallii* group genus, without a change in diversity after the intervention. According to the functional metabolic analysis by the Phylogenetic Investigation of Communities by Reconstruction of Unobserved States 2, the “Nitrate Reduction VI” and “Aspartate Superpathway” pathways were predicted to increase significantly in the fat loss group. The cooccurring networks of genera were constructed and showed the different microbes that drove the changes between the pre- and postintervention stages in the fat loss and fat gain groups.

**Conclusions:**

This study demonstrated that lifestyle modifications can impact the composition, richness, and predicted functional profiles of the gut microbiota in obese children after weight changes.

**Trial registration:**

ClinicalTrials.govNCT03812497, registration date January 23, 2019, retrospectively registered.

**Supplementary information:**

**Supplementary information** accompanies this paper at 10.1186/s12866-020-02002-3.

## Background

The prevalence of pediatric obesity has increased over the past decade, leading to an increase in concomitant childhood health conditions, including type 2 diabetes mellitus, hypertension, dyslipidemia, fatty liver disease, and psychological problems [1]. The imbalance between energy intake and expenditure is considered the most important cause of obesity [2]. The cornerstone of pediatric obesity management is lifestyle intervention, including dietary modification and increased physical activity, and not weight loss medicines, calorie-restricted diets, or bariatric surgery, which are current treatments for obese adults [3, 4].

Several studies have shown that obesity is associated with gut microbial dysbiosis [5, 6]. The contribution of the microbiome to obesity has been considered using multifactorial approaches, such as supplying additional calories to the host, affecting satiety, favoring fat storage, and disrupting the integrity of the epithelial barrier [7]. Recently, many studies, regarding weight loss interventions in adults, have investigated the relationship between the gut microbiota and obesity [8]. Some reports have demonstrated that weight reduction by bariatric surgery partially reversed obesity-associated microbial alterations in obese adults [9, 10]. A randomized controlled trial involving adults showed that modification of the gut microbiota composition by probiotics could reduce body weight [11, 12]. Regarding childhood obesity, most previous studies of the gut microbiota have been cross-sectional in design [13, 14]. A recent study in obese children revealed that secreted proteins of the gut microbiota affect the microbial composition [15]. One prospective study of children that investigated the microbial changes with weight gain over a four-year period showed that the microbiome-host-diet configuration could be a possible predictor of obesity [16]. However, research on microbial changes over time after weight reduction interventions has remained scarce [13, 17].

In this study, we aimed to investigate changes in the composition, diversity, predicted functional metabolic profiles, and correlation networks of the gut microbiota in obese children after lifestyle modifications.

## Results

After screening, 42 obese children participated in the first intervention, and six obese children declined further participation. In this study, adiposity was defined as the measured total body fat percentage in body composition analysis. A total of 36 participants in all of the interventions were classified into two groups: the fat loss group (*n* = 17, 47.2%), including those who experienced a decrease in total body fat (%) after the intervention, and the fat gain group (*n* = 19, 52.8%), including those who experienced an increase in total body fat (%) after the intervention.

The mean ages of the children were 10.0 (SD: 2.4) years in the fat loss group and 10.3 (SD: 2.7) years in the fat gain group (t-test, *P* = 0.733), and 58% of participants were male in each group (chi-square test, *P* = 0.542, Table 1). Birthweight, delivery type, duration of the intervention, and numbers of exercises and nutritional counseling sessions were not significantly different between the fat loss and fat gain groups (Table 1). The questionnaire on general lifestyle and eating habits was developed for this study, and its contents are shown in Additional file 1. No significant differences were detected in the results of the questionnaires between the fat loss and fat gain groups (Table 1). As expected, the fat loss group showed significantly decreased total body fat (%), total body fat mass (kg), visceral fat area (cm^2^), and abdominal fat (%) in the body composition analysis, whereas the fat gain group showed significantly increased values after the interventions (paired t-test and Wilcoxon’s signed-rank test, *P* < 0.05, Table 2). Body mass index (BMI) was significantly decreased in the fat loss group and significantly increased in the fat gain group after lifestyle modifications (paired t-test, *P* < 0.05, Table 2). In the fat loss group, the level of alanine aminotransferase (ALT) was significantly decreased after the intervention (Wilcoxon’s signed-rank test, *P* = 0.022, Table 2). The insulin level was significantly decreased in the fat loss group; however, the homeostasis model assessment method-insulin resistance (HOMA-IR) levels were significantly increased in both groups (Wilcoxon’s signed-rank test, *P* < 0.05, Table 2). Comparison of the anthropometric measurements and blood biochemical profiles before the intervention showed no significant differences between the fat loss and fat gain groups (Table S1).

**Table 1:** Comparison between the Fat Loss and Fat Gain Groups in the Results of the Questionnaires

	Fat loss (*n* = 17)	Fat gain (*n* = 19)	*P*
Sex			0.542
Female: male	6 (35.3%): 10 (58.8%)	8 (42.1%): 11 (57.9%)	
Age (years)	10.0 ± 2.4	10.3 ± 2.7	0.733
Birthweight (kg)	3.5 ± 0.5	3.2 ± 0.5	0.117
Delivery type			0.167
Vaginal: cesarean	5 (29.4%): 12 (70.6%)	11 (57.9%): 8 (42.1%)	
Duration of intervention (day)	57.0 [55.0; 62.0]	56.0 [51.0; 69.5]	0.536
Number of exercise counseling sessions			0.165
One	1 (5.9%)	0 (0.0%)	
Two	1 (5.9%)	5 (26.3%)	
Three	15 (88.2%)	14 (73.7%)	
Number of nutritional counseling sessions			0.382
One	1 (5.9%)	0 (0.0%)	
Two	1 (5.9%)	3 (15.8%)	
Three	15 (88.2%)	16 (84.2%)	
What did you feed your baby in the first year?			0.747
Exclusive breastfeeding	7 (41.2%)	8 (42.1%)	
Exclusive formula feeding	2 (11.8%)	3 (15.8%)	
Mixed with predominant breastfeeding	4 (23.5%)	2 (10.5%)	
Mixed with predominant formula feeding	4 (23.5%)	6 (31.6%)	
How long do you study after school?			0.540
Less than 1 h	2 (11.8%)	4 (21.1%)	
1–2 h	3 (17.6%)	7 (36.8%)	
2–3 h	5 (29.4%)	4 (21.1%)	
3–4 h	5 (29.4%)	3 (15.8%)	
More than 4 h	2 (11.8%)	1 (5.3%)	
How long do you do perform regular exercise each day?			0.767
None	3 (18.8%)	4 (21.1%)	
30 min	2 (12.5%)	7 (36.8%)	
30 min-1 h	7 (43.8%)	5 (26.3%)	
1–2 h	4 (25.0%)	6 (31.6%)	
More than 2 h	0 (0.0%)	1 (5.3%)	
How long do you use electronic devices each day?			0.504
Less than 2 h	7 (41.2%)	11 (57.9%)	
More than 2 h	10 (58.8%)	8 (42.1%)	
Is there an easily accessible place to exercise?			1.000
Yes	14 (82.4%)	16 (84.2%)	
No	3 (17.6%)	3 (15.8%)	
How do you get to school?			0.833
On foot	12 (70.6%)	15 (78.9%)	
By bus	1 (5.9%)	1 (5.3%)	
By private car	4 (23.5%)	3 (15.8%)	
Do you eat breakfast?			0.308
Never	6 (35.3%)	2 (11.1%)	
2–3 times a week	0 (0.0%)	1 (5.6%)	
4–5 times a week	2 (11.8%)	3 (16.7%)	
Daily	9 (52.9%)	12 (66.7%)	
How long does it take to eat a meal?			0.224
5 min	0 (0.0%)	3 (16.7%)	
10 min	6 (35.3%)	5 (27.8%)	
15 min	8 (47.1%)	4 (22.2%)	
20 min	2 (11.8%)	5 (27.8%)	
More than 20 min	1 (5.9%)	1 (5.6%)	
How many times a week do you eat late-night snacks?			0.990
Never	8 (47.1%)	10 (52.6%)	
1–2 times a week	7 (41.2%)	7 (36.8%)	
3–4 times a week	1 (5.9%)	1 (5.3%)	
Daily	1 (5.9%)	1 (5.3%)	
How many bottles of sugar-sweetened beverages do you drink a week?			0.537
None	2 (11.8%)	0 (0.0%)	
1 L bottle	7 (41.2%)	7 (46.7%)	
2 L bottle	6 (35.3%)	5 (33.3%)	
3 L bottle	2 (11.8%)	3 (20.0%)	

Data are expressed as counts (%)

**Table 2:** Comparison of the Results of Anthropometric Measurements and Blood Biochemical Profiles between the Pre- and Postintervention Stages in the Fat Loss and Fat Gain Groups

	Fat loss group		Fat gain group	
	pre (*n* = 17)	post (*n* = 17)	pre (*n* = 19)	post (*n* = 19)
Anthropometric measurements				
Weight (kg)	57.50 ± 16.90	57.38 ± 16.72	57.60 [49.40; 67.70]	60.20 [50.00; 68.90]^**^
Weight (z-score)	2.38 ± 0.76	2.29 ± 0.77^**^	2.16 [1.93; 2.50]	2.20 [1.94; 2.64]
Height (cm)	145.76 ± 14.80	146.78 ± 14.59^**^	148.73 ± 14.62	149.75 ± 14.59^**^
Height (z-score)	0.98 [0.17; 2.16]	1.04 [0.18; 2.17]	1.26 [0.75;1.46]	1.15 [0.86; 1.48]
BMI (kg/m^2^)	26.41 ± 4.04	26.01 ± 4.00^**^	25.70 [23.75; 27.30]	26.14 [23.94; 28.13]^*^
BMI (z-score)	2.54 [1.95; 2.76]	2.36 [1.88; 2.65]^**^	2.22 [1.96; 2.54]	2.34 [2.02; 3.54]
Systolic blood pressure (mmHg)	119.71 ± 13.74	118.18 ± 9.34	121.05 ± 11.71	119.26 ± 11.96
Diastolic blood pressure (mmHg)	77.00 [70.00; 82.00]	76.00 [69.00; 83.00]	72.05 ± 10.31	73.63 ± 10.72
Waist circumference (cm)	88.90 [75.00; 93.20]	84.50 [74.80; 93.90]^**^	88.81 ± 13.26	90.07 ± 13.64^*^
Waist-to-height ratio	0.58 [0.54; 0.61]	0.56 [0.53; 0.60]^**^	0. 59 [0.55; 0.62]	0.59 [0.56; 0.62]
Total body fat (%)	38.30 [35.60; 43.0]	37.2 [34.40; 39.70]^**^	38.79 ± 5.16	39.34 ± 5.06^*^
Skeletal muscle mass (kg)	17.70 [13.90; 21.80]	18.20 [14.20; 26.90]^**^	17.80 [15.70; 22.70]	19.40 [16.00; 23.80]
Total body fat (kg)	22.80 ± 7.89	21.99 ± 7.40^**^	21.60 [18.80; 26.80]	23.20 [19.20; 28.20]^**^
Visceral Fat (cm^2^)	112.10 [74.30; 144.20]	105.10 [71.90; 136.80]^**^	118.76 ± 49.54	123.76 ± 49.10^**^
Abdomen fat (%)	0.85 ± 0.08	0.85 ± 0.08	0.86 ± 0.10	0.87 ± 0.09
Blood biochemical profiles				
Glucose (mg/dL)	98.00 [95.00; 103.00]	99.00 [98.00; 103.00]	104.00 [100.0; 106.0]	99.00 [95.50; 107.00]
AST (IU/L)	24.00 [21.00; 29.00]	24.00 [19.00; 26.00]	24.000 [20.50; 30.50]	24.00 [20.00; 28.50]
ALT (IU/L)	20.0 [17.00; 35.00]	18.00 [16.00; 24.00]^*^	25.00 [15.00; 46.50]	26.00 [15.00; 42.50]
Total cholesterol (mg/dL)	177.00 ± 30.20	176.18 ± 31.84	169.58 ± 22.99	173.84 ± 18.90
Triglyceride (mg/dL)	90.00 [68.00; 117.00]	91.00 [67.00; 108.00]	77.00 [72.00; 115.00]	86.00 [64.00; 137.50]
HDL cholesterol (mg/dL)	50.94 ± 11.17	51.18 ± 10.67	52.47 ± 10.11	53.37 ± 10.87
LDL cholesterol (mg/dL)	108.29 ± 24.00	107.06 ± 27.04	102.32 ± 22.18	104.74 ± 19.33
hs-CRP (mg/L)	1.40 [0.60; 2.60]	1.40 [0.70; 1.90]	1.20 [0.80; 1.60]	1.60 [0.80; 2.00]
Uric acid (mg/L)	4.90 [4.40; 5.40]	4.80 [4.50; 5.50]	5.51 ± 0.93	5.79 ± 0.74
25-OH vitamin D (ng/mL)	16.08 ± 5.81	16.92 ± 6.44	13.63 ± 4.33	16.72 ± 5.92
Ferritin (ng/mL)	64.50 [48.30; 83.70]	68.70 [51.90; 96.20]	54.90 [41.40; 139.60]	59.80 [46.55; 111.75]
Insulin (μU/mL)	15.54 [10.75; 27.49]	11.63 [8.17; 16.12]^**^	19.29 [11.90; 39.21]	13.93 [10.43; 22.91]^**^
HbA1c (%)	5.4 ± 0.3	5.4 ± 0.2	5.40 [5.35; 5.50]	5.40 [5.30; 5.50]
HOMA-IR	1.33 [1.22; 1.42]	2.67 [2.05; 3.84]^**^	1.39 [1.33; 1.46]	2.99 [2.60; 6.17]^**^

Data are expressed as the means ± standard deviations or medians (interquartile ranges). ^*^P < 0.05,^**^P < 0.01 paired t-test or Wilcoxon’s signed-rank test between the pre- vs postintervention stages in the fat loss and fat gain groups. Abbreviations: *BMI* Body mass index; *AST* Aspartate aminotransferase; *ALT* Alanine aminotransferase; *HDL* High-density lipoprotein; *LDL* Low-density lipoprotein; *hs-CRP* High-sensitivity C-reactive protein

**Fig. 1:**
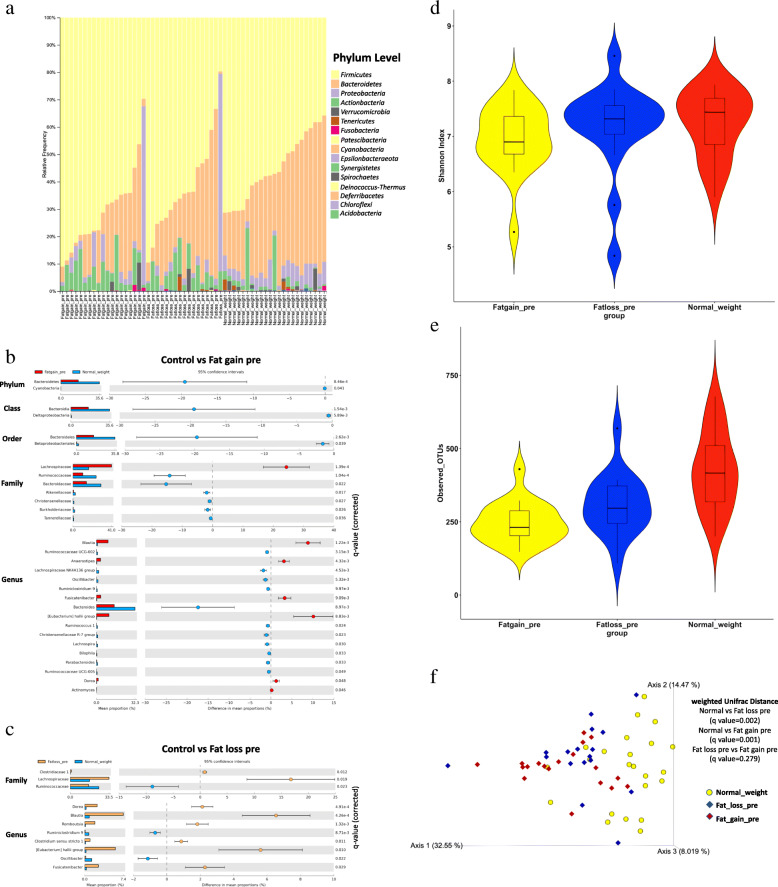
Baseline analyses of the composition and diversity of the gut microbiota in the controls and preintervention stages in the fat loss and fat gain groups. Bar plot represents the relative abundances of phylum in the controls, fat loss, and fat gain groups (**a**). Extended error bar plot for comparing the relative abundance of taxa at each phylogenetic level between the controls and preintervention stages in the fat gain (**b**) and fat loss groups (**c**). Violin plot for comparing the Shannon diversity index (**d**) and observed_OTUs index (**e**). Principal coordinate analysis based on weighted Unifrac distance (f)

For the baseline analysis, 16S rRNA gene sequencing data from the feces of the obese groups at the preintervention stages were compared with those of 24 normal-weight children (18 boys and 6 girls, aged 8.1 ± 1.5 years old) from our previous cross-sectional study of pediatric obesity as controls using Quantitative Insights into Microbial Ecology 2 (QIIME2) [18]. Among the results of anthropometric measurements, BMI in the obese group before intervention was significantly higher than that in the control group (Wilcoxon’s rank-sum test, *P* < 0.05, Table S2). A total of 8,221,270 sequences (mean of 139,343 sequences) were generated from 60 samples. After quality control, the dataset was reduced to a total of 6,074,850 sequences, with a mean of 102,963 sequences per sample, for 13,431 features. At the phylum level, Firmicutes and Bacteroidetes were dominant components of the gut microbiota, followed by Proteobacteria, Actinobacteria, and Verrucomicrobia in the fat loss, fat gain, and control groups (Fig. 1a). We compared the relative abundances of taxa between the control and the preintervention gut microbiota in the obese group using Statistical Analysis of Metagenomic Profiles (STAMP) software [19]. The relative abundance of Bacteroidetes was significantly lower in the preintervention stage in the fat gain group than in the control group (Welch’s t-test, FDR = 0.000846, Fig. 1b). The compositional differences in the gut microbiota of the controls and the preintervention stages in both groups at each phylogenetic level are shown in Fig. 1b. At the genus level, in the preintervention stage, both groups showed increased relative abundances of *Blautia*, *Dorea*, *Eubacterium hallii* group, and *Fusicatenibacter* compared with the control group, and the fat gain group also showed decreased proportions of *Bacteroides, Oscillibacter*, and *Parabacteroides* (Welch’s t-test, FDR < 0.05, Fig. 1b, c). No significantly different taxa at any phylogenetic level were identified between the fat loss and fat gain groups at the preintervention stage. The alpha rarefaction plot of the observed_Operational Taxonomic Unit (OTU) s indices presents the richness of the samples in the control and the preintervention stages in both groups (Fig. S1a). The Shannon diversity index showed no significant differences between the controls and the preintervention stages in both groups (Fig. 1d). In the preintervention stage, both groups showed significantly lower observed_OTUs indices than the control group, and the fat gain group showed significantly lower observed_OTUs indices than the fat loss group (Wilcoxon’s rank-sum test, controls vs fat loss pre q = 0.009, controls vs fat gain pre q = 0.00006, fat loss pre vs fat gain pre q = 0.022, Fig. 1e). Principal coordinate analysis (PCoA) based on the weighted Unifrac distances between the genus-level microbial profiles showed a significant separation between the controls and obese individuals in both groups by permutational multivariate analysis of variance (PERMANOVA) (controls vs fat loss pre: q = 0.002, controls vs fat gain pre: q = 0.001, fat loss pre vs fat gain pre: q = 0.279, Fig. 1f).

**Fig. 2:**
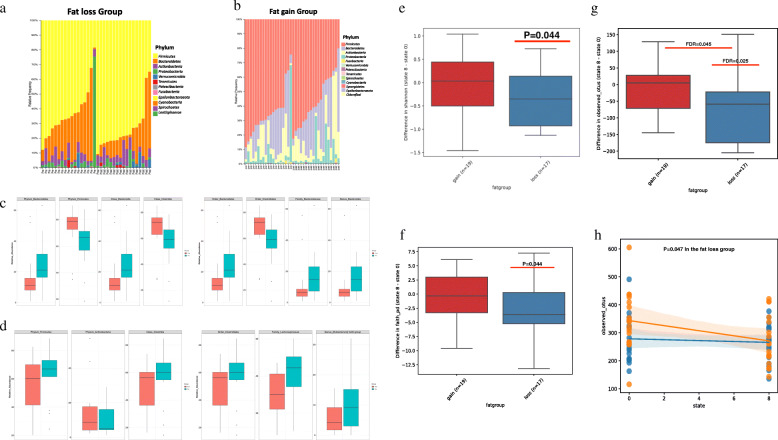
Comparisons of composition and diversity in the gut microbiota in the pre- and postintervention stages between the fat loss and fat gain groups. Bar plots represent the relative abundances of phylum in the fat loss (**a**) and fat gain (**b**) groups. Box plot for comparing the relative abundance of taxa at each phylogenetic level between the pre- and postintervention stages in the fat loss (**c**) and fat gain (**d**) groups. Diversity analysis of the gut microbiota in the fat loss and fat gain groups, including Shannon (**e**), Faith’s PD (f), and observed_OTUs (**g**) indices. Linear mixed effect model for observed_OTUs index in the fat loss and fat gain groups (**h**)

The 16S rRNA sequence datasets were collected from feces of the participants before and after the lifestyle modifications. A total of 10,895,632 sequences (mean of 151,328 sequences) were generated from 72 samples. After quality control, the dataset was reduced to a total of 7,296,519 sequences, with a mean of 99,854 sequences per sample, for 9617 features. At the phylum level, Firmicutes and Bacteroidetes were major components of the gut microbiota, followed by Actinobacteria, Proteobacteria, and Verrucomicrobia, in both the pre- and postintervention stages in the fat loss and fat gain groups (Fig. 2a, b). To investigate the changes in the microbial community after intervention, we performed pairwise differential abundance comparison with Analysis of Differential Abundance Taking Sample Variation Into Account (ALDEx2) (Bioconductor v.3.11) [20]. In the fat loss group, a total of 8 taxa (2 phyla, 2 classes, 2 orders, 1 family, and 1 genus) showed significantly different relative abundances after intervention (Wilcoxon signed-rank test, *P* < 0.05, Fig. 2c). The relative abundance of Firmicutes was significantly increased in the fat loss group (Wilcoxon’s signed-rank test, *P* = 0.009); conversely, the relative abundance of Bacteroidetes was significantly decreased in the fat loss group after intervention (Wilcoxon’s signed-rank test, *P* = 0.014, Fig. 2c). Bacteroidia class, Bacteroidales order, *Bacteroidaceae* family, and *Bacteroides* genus were significantly decreased, and Clostridiales order and Clostridia class were significantly increased in the fat loss group (Wilcoxon’s signed-rank test, *P* < 0.05, Fig. 2c). In the fat gain group, 6 taxa (2 phyla, 1 class, 1 order, 1 family, and 1 genus) showed significantly different relative abundances after intervention (Wilcoxon’s signed-rank test, *P* < 0.05, Fig. 2d). The relative abundance of Firmicutes was significantly decreased (Wilcoxon’s signed-rank test, *P* = 0.028) and the relative abundance of Actinobacteria was significantly increased in the fat gain group (Wilcoxon’s signed-rank test, *P* = 0.047, Fig. 2d). The proportions of Clostridia class, Clostridiales order, *Lachnospiraceae* family, and *Eubacterium hallii* group genus were significantly decreased in the fat gain group after intervention (Wilcoxon’s signed-rank test, *P* < 0.05, Fig. 2d).

The alpha rarefaction plot of the observed_OTUs indices clearly illustrates the richness of the samples in the pre- and postintervention stages in both groups (Fig. S1b). In the q2 longitudinal command in QIIME2, the alpha diversity indices, including the Shannon (Fig. 2e), Faith’s PD (Fig. 2f), and observed_OTUs (Fig. 2g) indices, were significantly changed in the fat loss group (Wilcoxon’s signed-rank test, *P* < 0.05) but not in the fat gain group. The degree of difference of the observed_OTUs indices after intervention was significantly higher in the fat loss group than in the fat gain group (Wilcoxon’s rank-sum test, FDR = 0.045, Fig. 2g). In the linear mixed-effect models, the observed_OTUs index was significantly decreased after lifestyle intervention in the fat loss group (coefficient: -7.5 [IQR: − 14.789, − 0.102], SD; 3.747, *P* = 0.047, Fig. 2h). The weighted Unifrac distance showed no significant differences between the pre- and postintervention stages in both groups (Fig. S2).

**Fig. 3:**
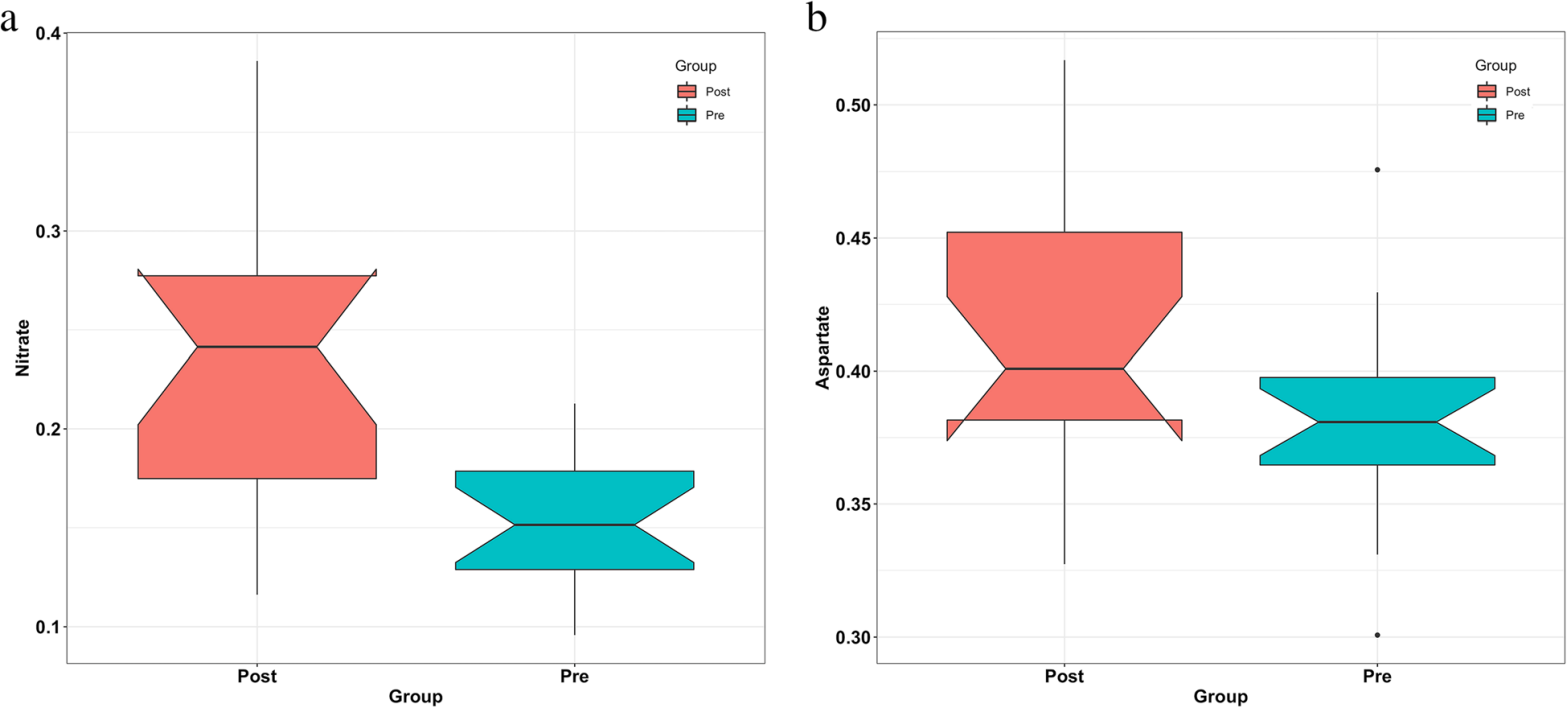
Functional profile analysis of the gut microbiota on the basis of the MetaCyc database in the fat loss group. The “Nitrate Reduction VI” (**a**) and “Aspartate Superpathway” (**b**) were predicted to increase after intervention in the fat loss group

A total of 346 and 363 metabolic pathways in the MetaCyc database were predicted by Phylogenetic Investigation of Communities by Reconstruction of Unobserved States (PICRUSt)2 in the fat loss and fat gain groups, respectively [21, 22]. The weighted nearest sequenced taxon index (NSTI) was used in the fat loss (mean ± SD: 0.127 ± 0.015) and fat gain (mean ± SD: 0.109 ± 0.010) groups [23]. To identify significantly changed predicted pathways in the microbial community after intervention, we performed pairwise differential abundance comparisons with ALDEx2. At the FDR 0.5 level, the “Nitrate Reduction VI” and “Aspartate Superpathway” metabolic pathways were predicted to significantly increase after intervention (Wilcoxon’s signed-rank test, FDR = 0.013, and 0.030, respectively, Fig. 3a, b). In the fat gain group, no significantly changed predicted pathways were identified.

**Fig. 4:**
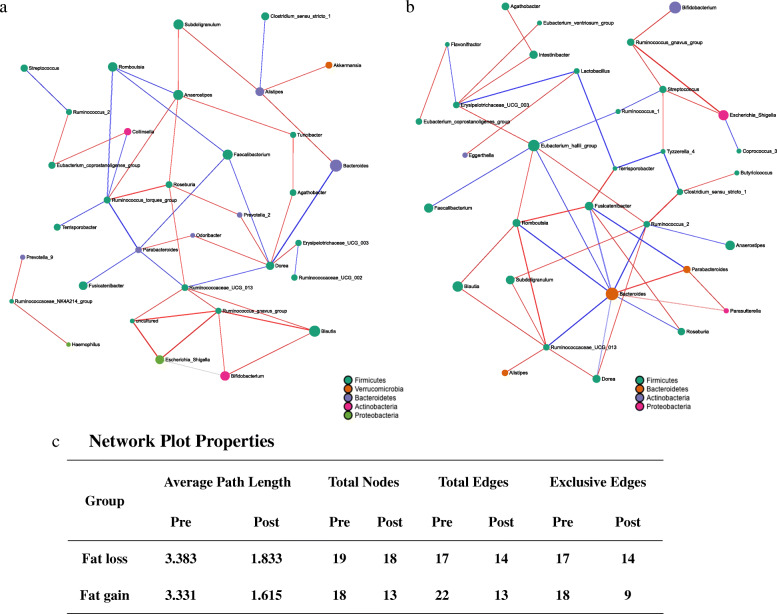
SparCC network plots of correlations between genus in the fat loss (**a**) and fat gain (**b**) groups. Nodes represent the amplicon sequence variant (ASV) at the genus level, with their size reflecting the ASVs’ average proportion in the community. The nodes are colored on the basis of phylum. Edges represent correlations between connecting nodes, with edge thickness indicating the correlation value and green and red colors indicating positive and negative correlations, respectively. Only significant correlations (two-sided *P* < 0.05 based on permutations of 100 iterations) with correlation thresholds > 0.3 are presented. Network plot properties are presented (**c**)

**Fig. 5:**
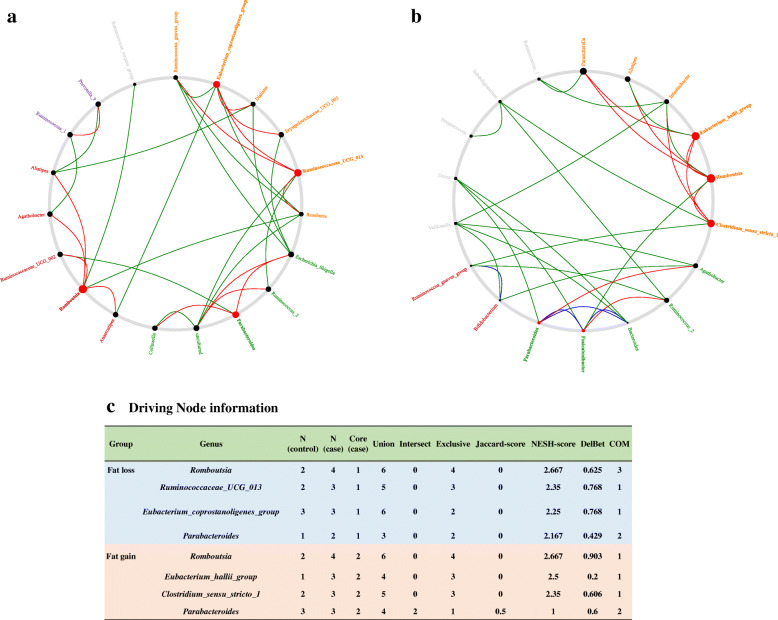
The driving genus that drove the changes between the pre- and postintervention stages in the fat loss group (**a**) and in the fat gain group (**b**). Red nodes represent the drivers identified by the NetShift method. The sizes of nodes are proportional to the NESH scores, and the nodes that are colored red have increased betweenness from the prestage to the poststage. The large red nodes are considered drivers. Summary of the node properties in both groups (**c**)

Analyses of the cooccurrence networks of genera in the fat loss and fat gain groups were conducted (Fig. 4a, b). Nodes represent the Amplicon Sequence Variant (ASV) s at the genus level, with size reflecting the relative abundance in the community and color indicating the phylum. The edge represents the correlation between connecting nodes, with edge thickness indicating the correlation value and green and red colors indicating positive and negative correlations, respectively. Only significant correlations (two-sided pseudo *P* < 0.05 based on permutations of 100 iterations) with correlation thresholds ≥ 0.3 are presented. The microbial dysbiosis index (MD index) was 0.6009 in the pre/postintervention fat loss group and 0.9251 in the pre/postintervention fat gain group [24]. Regarding the network plot properties, the average path length, total nodes, and total edges decreased after the lifestyle intervention (Fig. 4c). On the basis of NetShift analysis, the genera *Romboutsia*, *Ruminococcaeceae*_*UCG_013*, *Eubacterium coprostanollgenes-group*, and *Parabacteroides* were identified as driver genera, with key roles in the changes in microbial interactions during the intervention in the fat loss group (Fig. 5a, c). We were also able to identify the genera *Romboutsia*, *Eubacterium_halli_*group, and *Clostridium_sensu_stricto*_1 as driving the microbial community changes from the pre- to the postintervention stages in the fat gain group (Fig. 5b, c).

## Discussion

The main treatment for obesity in children includes lifestyle modifications, such as dietary modification, increased physical activity, and behavioral modification, rather than medications or bariatric surgery [25]. In recent decades, growing evidence has identified an association between the gut microbiota and obesity, and microbiota-targeted strategies have attracted attention in the context of obesity treatment [26]. This study demonstrated that lifestyle modifications could exert a significant influence on the composition, diversity, and predicted functional profiles of the gut microbiota in childhood obesity.

In the baseline analysis, the obese children in the fat gain group showed decreased proportions of Bacteroidetes compared with the controls, as described by previous studies [15, 18, 27, 28]. The gut microbiota in the obese children showed a different microbial composition from that in the controls, including increased proportions of the genera *Blautia*, *Dorea*, *Eubacterium hallii* group, and *Fusicatenibacter*, which were reported to be associated with obesity, as well as decreased proportions of the genera *Bacteroides, Oscillibacter*, and *Parabacteroides*, which were positively associated with leanness [18, 29–31]. In both the preintervention groups, reduced microbial richness was noted, and the beta diversity showed significant separation between the control and obese groups, generally implicating dysbiotic features [30].

Recent studies have reported that the microbiome adapts quickly to lifestyle changes [32]. In the fat loss group, the relative abundances of Firmicutes and Bacteroidetes were changed significantly after lifestyle modifications. Previous clinical weight reduction trials reported no consistent results regarding these major phyla. Some trials showed an increase in the relative abundance of Bacteroidetes along with a decrease in Firmicutes; others reported the opposite effects on these phyla, similar to the results of our study, while a few trials observed no impact [13, 33–35]. A population-based study of school-aged children showed that these inconsistent results in microbial communities are related to environmental factors, including socioeconomic status, age, and weight [36]. The members of Bacteroidetes were reported to be more largely influenced by environmental factors rather than by host genetics, and some clinical trials demonstrated that these members are associated positively with a diet rich in protein and animal fat [16, 37, 38]. Our study demonstrated that Bacteroidia class, Bacteroidales order, *Bacteroidaceae* family, and *Bacteroides* genus, belonging to the Bacteroidetes phylum, were significantly decreased in the fat loss group. This decrease could be related to diet changes, including a decrease in highly processed foods rich in animal fat for 2 months. Otherwise, among the members of Firmicutes, increased proportions of Clostridiales order and Clostridia class in the fat loss group as well as decreased proportions of Clostridia class, Clostridiales order, *Lachnospiraceae* family, and *Eubacterium hallii* group genus in the fat gain were demonstrated after intervention. One possible explanation for these changes in the relative abundance of Firmicutes is that our lifestyle modification program did not include a carbohydrate-restrictive diet. A carbohydrate-restrictive diet can result in a reduction in the abundance of Firmicutes due to low intake of complex carbohydrates, which act as prebiotics [39]. Further studies are required to determine whether the changes in microbiota composition were the result of lifestyle modifications.

In the fat loss group, the bacterial taxa richness was decreased after the application of lifestyle modifications. This observation is contrary to previous studies, which showed that a diet intervention, including calorie and carbohydrate restriction, increases in microbial gene richness with a decrease of adiposity in participants [40, 41]. However, some studies in adults have revealed that microbial richness decreased after a short-term dietary restriction, and other studies in pediatric populations showed no differences [42–44]. A recent study in Mexican children revealed significantly greater richness and diversity in the obese group than in the normal-weight group [15]. In one study of 61 adults who underwent bariatric surgery for obesity treatment, low microbial gene richness was correlated with truncal fat mass and remained low at 1 year after surgery, suggesting a complex interplay between the gut microbiome and host obesity [10]. Shoaie et al. analyzed the correlations of reduced bacterial gene counts and the production of several amino acids with the occurrence of metabolic diseases using a computational tool, and they showed that dietary interventions might reduce these products and improve insulin sensitivity [45]. The microbial richness in obesity with weight changes shows an inconsistent pattern, suggesting that more studies are required.

The mechanism by which the microbiota affects energy balance in the human body are not clear. Our results from the PICRUSt2 analysis demonstrated two metabolic pathways in the MetaCyc database that were predicted to significantly increase after lifestyle modifications in the fat loss group. In the “Nitrate Reduction VI” pathway, the final product is L-glutamine, supplementation with which has recently been reported to lead to weight loss [46, 47]. The “Aspartate Superpathway” pathway includes both L-aspartate biosynthesis and degradation to oxaloacetate due to reversible transamination. Oxaloacetate was reported to activate brain mitochondrial biogenesis, leading to enhancement of the insulin pathway [48]. However, these predictions based on the 16S rRNA gene amplicon sequencing data could be limited because the actual functions of the full metagenome likely differ, although amplicon-based predictions might be highly correlated with functional profiles based on shotgun metagenomics sequencing data [49]. To increase the accuracy of predictions, we used the weighted NSTI value, which is a measure of how closely related the ASVs in each sample are to the reference genomes in the database [50]. Analysis was conducted after excluding ASVs presenting weighted NSTI values of 0.15 or greater, indicating generally unreliable prediction [49]. Much more research is required to determine the specific mechanisms associated with functional analysis.

We analyzed the microbial cooccurrence network with Sparse Correlation for Compositional data (SparCC) analysis, which is capable of avoiding the microbial compositional bias introduced when correlating relatively abundant data in Spearman’s and Pearson’s analyses [51]. The MD index in SparCC is the log ratio of the total abundance of genera increased in the preintervention group to the total abundance of genera decreased in the postintervention group [24]. In the previous study, this index showed a strong positive correlation with clinical disease severity and a negative correlation with species richness, resulting in an empirical estimation of the degree of dysbiosis within the microbiome [24]. The fat gain group had a higher MD index value than the fat loss group, representing dysbiosis or an imbalance in the microbial community after intervention. In the network plot properties, the degrees of decrease in the average path length, total nodes, and total edges in the postintervention stage compared to those in the preintervention stage in the fat gain group were greater than those in the fat loss group. This outcome suggests that the relationships of the gut microbiota in the fat gain group decreased to be fewer than those in the fat loss group after intervention.

In the NetShift analysis, the driver genera that played key roles in microbial interactions were different between the fat loss and fat gain groups. The role of *Parabacteroides* as a driver of community change after lifestyle intervention was greater in the fat loss group than in the fat gain group. This finding suggested that *Parabacteroides* might play a key role in the process of weight loss during lifestyle modifications. Recent studies have revealed that *Parabacteroides* exerts an anti-obesogenic effect and could suppress the systemic inflammatory response by regulating IL-10 and Treg cells [31, 43]. Another driver taxon in the fat loss group was *Ruminococcaceae_UCG_013*, which was demonstrated to be associated with a decreased risk of weight gain, suggesting functional linkage to a lean phenotype in a large-scale, longitudinal adult study [52]. Another driver genus in the fat loss group was *Eubacterium_coprostanoligenes*_group, which is known to reduce cholesterol by cholesterol-to-coprostanol conversion [53]. The only common driver taxon between the fat loss and fat gain groups was *Romboutsia*, which was recently described [54]. *Romboutsia* produces end products such as acetic acid, ethanol, iso-butanoic acid and iso-valeric acid, which are substrates involved in gluconeogenesis and lipogenesis [55]. Other driver taxa in the fat gain group were *Eubacterium hallii* and *Clostridium* sensu stricto groups, which produce short-chain fatty acids [56, 57]. These results suggest that lifestyle modifications might exert a different effect on the interactions between microbial communities according to the direction of weight change.

In adult studies, changes in the gut microbiota were associated with weight reduction interventions, including restrictive diets, bariatric surgery, and medications including pre, pro-, and synbiotics and metformin; these interventions are difficult to apply in children due to the risk of nutritional imbalance and surgery [58]. Our study showed BMI reduction in approximately 50% of participants; this result was relatively higher than those in adult studies involving weight loss interventions, which have generally resulted weight loss in 5–20% of participants [59, 60]. Our weight reduction program was a multidisciplinary individualized approach with frequent contact, which is the principal treatment of pediatric obesity, rather than restricted diet or surgery. Frequent management seemed to be effective in reducing the weight of the participants in our study. Most previous cross-sectional studies of childhood obesity have reported altered gut microbiota; however, few clinical trials have investigated gut microbial changes with weight reduction interventions [13, 14]. One study in preschool children participating in a behavioral intervention program reported no significant changes in microbial composition or functional profiles associated with weight loss [13]. However, our study showed altered gut microbial composition, richness, and predicted functional profiles with weight loss. These inconsistent results could be related to the report that the gut microbiota varies with age, ethnicity and diet [36]. Our study of Korean obese children could provide additional valuable information on common traits characterizing pediatric obesity.

Among the markers of the insulin resistance of the fat loss group, the fasting insulin level was improved after the intervention, although HOMA_IR was increased. One possible explanation is that the increase of HOMA-IR levels could be associated with unchanged fasting glucose and HbA1C levels, because our intervention included no carbohydrate restriction. Some studies have reported limitations of HOMA-IR in subjects with high fasting glucose levels and indicated that the fasting insulin value, rather than HOMA-IR, could be a surrogate measure of insulin resistance [61, 62].

Although the duration of the intervention, number of exercises, and nutritional counseling were not significantly different between the fat loss and fat gain groups, the responses to the intervention were different. In this regard, some considerations were noted in our study. In the baseline study, higher microbial richness was identified in the fat loss group than in the fat gain group. This observation agrees with other studies revealing that higher bacterial richness was associated with greater decreases in adiposity for obese adults with bariatric surgery and dietary interventions [10, 42]. The microbial richness at baseline might be a predictive potential factor for the efficacy of interventions. Further, a recent study revealed that baseline microbiota composition is not predictive of weight loss for the intervention [32]. In line with this finding, our baseline study showed no significant differences in the microbial composition between the fat loss and fat gain groups. One limitation of our study was the small number of participants. Another limitation is that we cannot identify whether the gut microbiota will recover and how long it will take. To clarify the function and pathophysiology of the gut microbiota in relation to childhood obesity, further trials that include a larger number of obese children are necessary.

## Conclusions

In conclusion, we observed significant alterations in the composition, richness, and expected functional profiles of the gut microbiota with weight loss after lifestyle modifications. Lifestyle modifications could impact microbiota dynamics, although little is known about the effect in obese children.

## Methods

### Participants, questionnaires, and anthropometric measurements

This longitudinal cohort study was an analysis of fecal samples collected from obese children before and after a 2-month weight reduction program. We recruited 50 obese children aged 7 to 18 years old at Hallym University Kangnam Sacred Heart Hospital from August 2018 to August 2019. Obesity was defined as a BMI ≥ 95th percentile based on the 2017 Korean growth chart [63]. Those who had congenital heart disease, chronic inflammatory bowel disease, chronic liver disease, or chronic renal disease were excluded. The participants were required to take no antibiotics, probiotics, or steroids for 1 month prior to the intervention. If participants had acute inflammatory diseases, such as influenza, pneumonia, or acute gastroenteritis, we delayed the lifestyle modification program by 1 month. The participants completed questionnaires, which provided multiple choice questions on general lifestyle (the time spent studying and using electronic devices, the duration and frequency of regular exercise, the presence of easily accessible locations to exercise, and their mode of transportation to school) and eating habits (meal duration, the consumption of late-night snacks, the consumption of breakfast, and the intake of sugar-sweetened beverages) and submitted them at the first hospital visit. Anthropometric measurements, including height, weight, waist circumference, and blood pressure, were performed by professionally trained research nurses at the first and third hospital visits [64, 65]. Body composition analysis of total body fat mass, skeletal muscle mass, total body fat percent, visceral fat area, and abdominal fat percent was performed with an InBody 770 analyzer (Biospace Co. Ltd., Seoul, South Korea) at the first and third hospital visits.

### Weight reduction programs

Over 8 weeks, the participants received weight reduction counseling from dietitians, exercise professionals, research nurses, and a pediatric clinician three times. At the first visit, the pediatric clinician, who specialized in childhood obesity, designed individualized feasible lifestyle modification programs in conjunction with dietitians, exercise professionals, and research nurses based on interviews with the participants and their guardians. Dietitians provided practical nutritional counseling on eating habits rather than a restrictive meal menu, and they suggested one or two individualized “must-follow” recommendations, such as eating breakfast, avoiding sugar-sweetened beverages, decreasing processed foods rich in animal fat or lengthening meal duration, to the participants at every counseling session. Exercise professionals found ways for obese children to exercise without a disruption to their general lives after analyzing the participants’ lifestyles, and they suggested one or two individualized “must-follow” recommendations, such as performing daily stretching exercises at home, cycling after school or on the weekends, using stairs instead of elevators if safe, and walking to school or academy, to the participants at every counseling session. The research nurse monitored the children to confirm that they were following the recommendations provided every 2 weeks. At the third visit, the pediatric clinician interviewed the participants and their guardians and suggested practical ways for participants to maintain their “must-follow” recommendations in general life.

### Blood sampling and biochemical analysis

At the first and third visits, blood samples were obtained from the participants after an 8-h overnight fast. The levels of glucose, aspartate aminotransferase (AST), ALT, total cholesterol, triglycerides, high-density lipoprotein (HDL) cholesterol, low-density lipoprotein (LDL) cholesterol, high-sensitivity C-reactive protein (hs-CRP) and uric acid were measured using a Hitachi 7600 autoanalyzer (Hitachi, Tokyo, Japan). Concentrations of ferritin, insulin, and 25-OH vitamin D were determined using an ADVIA Centaur XP instrument (Siemens Diagnostics, Deerfield, IL, USA). The hemoglobin A1c (HbA1c) level was determined using a D-100 system (Bio-Rad Laboratories, Hercules, CA, USA). HOMA-IR was calculated as [insulin (μIU / mL) × glucose (mg / dL)]/ 405.

### 16S rRNA gene amplicon sequencing using an Illumina MiSeq platform and bioinformatics analysis

Fecal samples were collected before and after the interventions. Fecal samples were subsequently stored at − 80 °C within 1 h of sampling until DNA extraction. The genomic DNA in fecal samples was extracted using a FastDNA™ SPIN Kit for Soil (MP Biomedicals, Santa Ana, CA, USA) according to the manufacturer’s instructions. The DNA concentration was measured using a Quant-iT™ PicoGreen™ dsDNA Assay Kit (Invitrogen, Waltham, MA, USA). 16S rRNA gene amplicon sequencing of the V3–4 regions was performed by a commercial company (Chunlab Inc., Seoul, South Korea) using an Illumina MiSeq platform (Illumina, San Diego, CA, USA) [66]. The sequencing outputs were generated as demultiplexed fastq sequences for downstream analysis using QIIME2, which identifies ASVs rather than OTUs. AVS approaches are generally considered to provide more precise identifications of microbes than OTU approaches [67]. The dada2 denoise-paired command was used to filter the low-quality and chimeric sequences in the fastq reads. The taxonomy of these features was assigned via the Silva-genes reference database classifier (version 136) considering 99% similarity. 16S rRNA gene sequencing data of the preintervention stages in both groups were compared with those of 24 normal-weight children in our previous cross-sectional study of pediatric obesity as controls [18]. The differential compositional analyses of the gut microbiota between the controls and both groups in the preintervention stages were performed by STAMP software [19]. Alpha diversity (observed_OTUs index and Shannon index) and beta diversity (weighted Unifrac distance) between the control and the preintervention stages of the obese groups were analyzed at the sequence depth of 5041 in QIIME2 and were visualized by the ggplot2 package in R software. Pairwise significant differences in the relative abundances at phylogenetic levels between the pre- and postintervention stages of both groups were assessed by the ALDEx2 package and were visualized by the ggplot2 package in R software. The q2 longitudinal command in QIIME2 was used to analyze pairwise diversity changes after the intervention at the sequence depth of 5100. Linear mixed-effect models were constructed to analyze the relationships of the statistically significant alpha diversity indices. The PICRUSt2 (v2.3.0 beta) tool was used to infer the functional potentials of the gut microbiota on the basis of Enzyme Classification (EC) numbers in the MetaCyc database from 16S rRNA gene amplicon sequencing [21, 22]. The ASV table generated from QIIME2 was rarefied at the sequence depth of 5100 and then applied to PICRUSt2. After transforming the relative abundance, the ALDEx2 package was used to identify the significantly changed predicted functional pathways in our data after the intervention.

SparCC analysis at the genus level was conducted to detect coabundance and coexclusion correlations with two-sided pseudo *P*-values (*P*-values < 0.05 considered significant) based on 100 iterations in the MicrobiomeAnalyst web application [68]. NetShift analyses were performed to detect the driver microbes in the web application based on the networks generated by SparCC [69].

### Statistical methods

Paired normally distributed data were analyzed using the paired t-test and are presented as the means and standard deviations; paired nonnormally distributed data were analyzed using the Wilcoxon’s signed-rank test and are presented as medians and interquartile ranges. The t-test and Wilcoxon’s rank-sum test were used to analyze the independent normally distributed data and the independent skewed continuous data, respectively. Categorical variables were analyzed by the chi-square test and are presented as frequencies and percentages. Statistical significance was declared at a *P*-value < 0.05. Resulting *P*-values were adjusted for multiple testing with the false discovery rate (FDR) method.

## Supplementary information

**Additional file 1.** Questionnaire on general lifestyle and eating habits.

**Additional file 2: Table S1**. Comparison of the Anthropometric Measurements and Blood Biochemical Findings between the Fat Loss and Fat Gain Groups in the Pre- and Postintervention Stages. **Table S2**. The Characteristics of the Normal Weight and the Preitervention Stages in the Fat Loss and Fat Gain Groups.

**Additional file 3: Fig. S1.** The alpha rarefaction plots for the observed_OTUs index in the baseline study, including the controls and preintervention stages of the fat loss and fat gain groups (a), and the intervention study, including the pre- and postintervention stages in the fat loss and fat gain groups (b). **Fig. S2.** Principal coordinate analysis based on the weighted Unifrac distance between the pre- and postintervention stages in the fat loss (a) and fat gain groups (b).

## Data Availability

All raw 16S rRNA gene sequencing data have been deposited in the NCBI Sequence Read Archive (SRA) under accession number SUB7459302 (BioProject PRJNA633584).

## Abbreviations

F B ratio: Firmicutes to bacteroidetes ratio
OTU: Operational taxonomic unit
ASV: Amplicon sequence variant
QIIME2: Quantitative insights into microbial ecology 2
STAMP: Statistical analysis of metagenomic profiles
ALDEx2: Analysis of differential abundance taking sample variation into account 2
PERMANOVA: Permutational multivariate analysis of variance
PICRUST2: Phylogenetic investigation of groups by reconstruction of unobserved states 2
SparCC: Sparse correlation for compositional data
