# Supplementary material for: Lifestyle modifications result in alterations in the gut microbiota in obese children
Source: BMC Microbiol. 2021 Jan 6;21:10. doi: 10.1186/s12866-020-02002-3 (PMC7789654; doi:10.1186/s12866-020-02002-3)
Supplement: Supplementary file 1 — Additional file 1. Questionnaire on general lifestyle and eating habits. [file 12866_2020_2002_MOESM1_ESM.pdf]

Questionnaires On General Lifestyle And Eating Habits

1. Name: _______________
2. Birth date: _______________, Age (   ) years old
3. Gender: Male (   ), Female (   )
4. Registration Date: ________________
5. Birth weight (kg): ______
6. Delivery type: Vaginal (   ), Cesarean (   )
7. What did you feed your baby in the first year? (   )
    A. Exclusive breastfeeding
    B. Exclusive formula feeding
    C. Mixed with predominant breastfeeding
    D. Mixed with predominant formula feeding
8. How long do you study after school? (   )
    A. Less than one hour
    B. 1-2 hours
    C. 2-3 hours
    D. 3-4 hours
    E. More than 4 hours
9. How long do you do perform regular exercise each day? (   )
    A. None
    B. 30 minutes
    C. 30 minutes - 1 hour
    D. 1-2 hours
    E. More than 2 hours
10. How long do you use electronic devices each day? (   )

A. Less than 2 hours

B. More than 2 hours

11. Is there an easily accessible place to exercise? (   )

A. Yes

B. No

12. How do you get to school? (   )

A. On foot

B. By bus

C. By private car

13. Do you eat breakfast? (   )

A. Never

B. 2-3 times a week

C. 4-5 times a week

D. Daily

14. How long does it take to eat a meal? (   )

A. 5 minutes

B. 10 minutes

C. 15 minutes

D. 20 minutes

E. More than 20 minutes

15. How many times a week do you eat late-night snacks? (   )

A. Never

B. 1-2 times a week

C. 3-4 times a week

D. Daily

16. How many bottles of sugar-sweetened beverages do you drink a week? (   )

A. None

B. 1 L bottle

C. 2 L bottle

D. 3 L bottle
