# Supplementary material for: Lifestyle modifications result in alterations in the gut microbiota in obese children
Source: BMC Microbiol. 2021 Jan 6;21:10. doi: 10.1186/s12866-020-02002-3 (PMC7789654; doi:10.1186/s12866-020-02002-3)
Supplement: Supplementary file 2 — Additional file 2: Table S1. Comparison of the Anthropometric Measurements and Blood Biochemical Findings between the Fat Loss and Fat Gain Groups in the Pre- and Postintervention Stages. Table S2. The Characteristics of the Normal Weight and the Preitervention Stages in the Fat Loss and Fat Gain Groups. [file 12866_2020_2002_MOESM2_ESM.docx]

Table S1. Comparison of the Anthropometric Measurements and Blood Biochemical Findings between the Fat Loss and Fat Gain Groups in the Pre- and Postintervention Stages.

|  | Preintervention | | Postintervention | |
| --- | --- | --- | --- | --- |
|  | Fat loss (n = 17) | Fat gain (n = 19) | Fat loss (n = 17) | Fat gain (n = 19) |
| Anthropometric measurements | | | | |
| Weight (kg) | 57.5 ± 16.9 | 61.0 ± 21.9 | 57.4 ± 16.7 | 62.5 ± 22.1 |
| Weight (z-score) | 2.3 [1.9; 2.8] | 2.2 [1.9; 2.5] | 2.1 [1.7; 2.7] | 2.2 [1.9; 2.6] |
| Height (cm) | 145.8 ± 14.8 | 148.7 ± 14.6 | 146.8 ± 14.6 | 149.8 ± 14.6 |
| Height (z-score) | 1.0 ± 1.0 | 1.3 ± 1.0 | 0.9 ± 1.0 | 1.3 ± 0.9 |
| BMI (kg/m^2^) | 26.1 [23.2; 28.0] | 25.7 [23.8; 27.3] | 26.0 ± 4.0 | 26.9 ± 5.3 |
| BMI (z-score) | 2.5 [1.9; 2.8] | 2.2 [2.0; 2.5] | 2.4 [1.9; 2.6] | 2.3 [2.0; 3.5] |
| Systolic blood pressure (mmHg) | 119.7 ± 13.7 | 121.1 ± 11.7 | 118.2 ± 9.3 | 119.3 ± 12.0 |
| Diastolic blood pressure (mmHg) | 75.9 ± 8.0 | 72.1 ± 10.3 | 75.3 ± 10.2 | 73.6 ± 10.7 |
| Waist circumference (cm) | 86.1 ± 11.4 | 88.8 ± 13.3 | 84.3 ± 10.8 | 90.1 ± 13.6 |
| Waist-to-height ratio | 0.6 [0.5; 0.6] | 0.6 [0.6; 0.6] | 0.6 [0.5; 0.6] | 0.6 [0.6; 0.6] |
| Total body fat (%) | 39.6 ± 5.1 | 38.8 ± 5.2 | 38.3 ± 5.0 | 39.3 ± 5.1 |
| Skeletal muscle mass (kg) | 18.4 ± 6.1 | 19.8 ± 7.8 | 18.2 [14.2; 26.9] | 19.4 [16.0; 23.8] |
| Total body fat (kg) | 22.8 ± 7.9 | 23.9 ± 9.6 | 22.0 ± 7.4 | 24.9 ± 10.0 |
| Visceral fat (cm^2^) | 114.8 ± 40.4 | 118.8 ± 49.5 | 108.5 ± 38.8 | 123.8 ± 49.1 |
| Abdominal fat (%) | 0.9 ± 0.1 | 0.9 ± 0.1 | 0.8 ± 0.1 | 0.9 ± 0.1 |
| Blood biochemical profiles | | | | |
| Glucose (mg/dL) | 98.0 [95.0; 103.0] | 104.0 [100.0; 106.0] | 99.0 [98.0; 103.0] | 99.0 [95.5; 107.0] |
| AST (IU/L) | 24.0 [21.0; 29.0] | 24.0 [20.5; 30.5] | 24.0 [19.0; 26.0] | 24.0 [20.0; 28.5] |
| ALT (IU/L) | 20.0 [17.0; 35.0] | 25.0 [15.0; 46.5] | 18.0 [16.0; 24.0] | 26.0 [15.0; 42.5] |
| Total cholesterol (mg/dL) | 177.0 ± 30.2 | 169.6 ± 23.0 | 176.2 ± 31.8 | 173.8 ± 18.9 |
| Triglyceride (mg/dL) | 90.0 [68.0; 117.0] | 77.0 [72.0; 115.0] | 91.0 [67.0; 108.0] | 86.0 [64.0; 137.5] |
| HDL cholesterol (mg/dL) | 50.9 ± 11.2 | 52.5 ± 10.1 | 51.2 ± 10.7 | 53.4 ± 10.9 |
| LDL cholesterol (mg/dL) | 102.3 ± 22.2 | 108.3 ± 24.0 | 107.1 ± 27.0 | 104.7 ± 19.3 |
| hs-CRP (mg/L) | 1.4 [0.6; 2.6] | 1.2 [0.8; 1.6] | 1.4 [0.7; 1.9] | 1.6 [0.8; 2.0] |
| 25-OH vitamin D (ng/mL) | 16.1 ± 5.8 | 13.6 ± 4.3 | 15.6 [13.1; 19.2] | 14.9 [13.0; 19.0] |
| Ferritin (ng/mL) | 64.5 [48.3; 83.7] | 54.9 [41.4; 139.6] | 68.7 [51.9; 96.2] | 59.8 [46.5; 111.8] |
| Insulin (µU/mL) | 15.5 [10.8; 27.5] | 19.3 [11.9; 39.2] | 11.6 [8.2; 16.1] | 13.9 [10.4; 22.9] |
| HbA1c (%) | 5.4 [5.2; 5.5] | 5.4 [5.3; 5.5] | 5.3 [5.2; 5.5] | 5.4 [5.3; 5.5] |
| HOMA-IR | 1.3 [1.2; 1.4] | 1.4 [1.3; 1.5] | 2.7 [2.0; 3.8] | 3.0 [2.6; 6.2] |
| Data are expressed as the means ± standard deviations or medians (interquartile ranges). | | | | |

| Table S2. The Characteristics of the Normal Weight and the Preintervention Stages in the Fat Loss and Fat Gain Groups. | | | | | |
| --- | --- | --- | --- | --- | --- |
|  | Fat_loss_pre (n = 17) | Fat_gain_pre (n = 19) | Normal_weight (n = 24) | P-value (nl_vs_loss) | P-value (nl_vs_gain) |
| Sex |  |  |  |  |  |
| Female: male | 7 (41.2%): 10 (58.8%) | 8 (42.1%): 11 (57.9%) | 6 (25%): 18 (75%) | 0.450 | 0.389 |
| Age (years) | 10.0 ± 2.4 | 10.1 [ 9.2; 11.2] | 8.1 ± 1.5 | 0.003^*^ | 0.001^*^ |
| Weight (kg) | 57.5 ± 16.9 | 61.0 ± 21.9 | 28.3 ± 6.7 | 0.000^*^ | 0.000^*^ |
| Weight (z-score) | 2.3 [1.9; 2.8] | 2.2 [ 1.9; 2.5] | 0.5 [ 0.3; 1.0] | 0.000^*^ | 0.000^*^ |
| Height (cm) | 145.8 ± 14.8 | 148.7 ± 14.6 | 129.5 ± 9.4 | 0.001^*^ | 0.000^*^ |
| Height (z-score) | 1.0 ± 1.0 | 1.3 ± 1.0 | 0.6 ± 0.5 | 0.194 | 0.005^*^ |
| BMI (kg/m^2^) | 26.1 [23.2; 28.0] | 25.7 [23.8; 27.3] | 16.6 [15.5; 17.6] | 0.000^*^ | 0.000^*^ |
| BMI (z-score) | 2.5 [ 1.9; 2.8] | 2.2 [ 2.0; 2.5] | 0.6 [ 0.4; 0.9] | 0.000^*^ | 0.000^*^ |
| Data are expressed as the means ± standard deviations or counts (%). ^*^P < 0.05. Abbreviations: nl; the normal-weight group, loss; the preintervention stage of the fat loss group; gain: the preintervention stage of the fat gain group. | | | | | |
