# Supplementary figures and images for: Lifestyle modifications result in alterations in the gut microbiota in obese children
Source: BMC Microbiol. 2021 Jan 6;21:10. doi: 10.1186/s12866-020-02002-3 (PMC7789654; doi:10.1186/s12866-020-02002-3)

**Fig S1a**

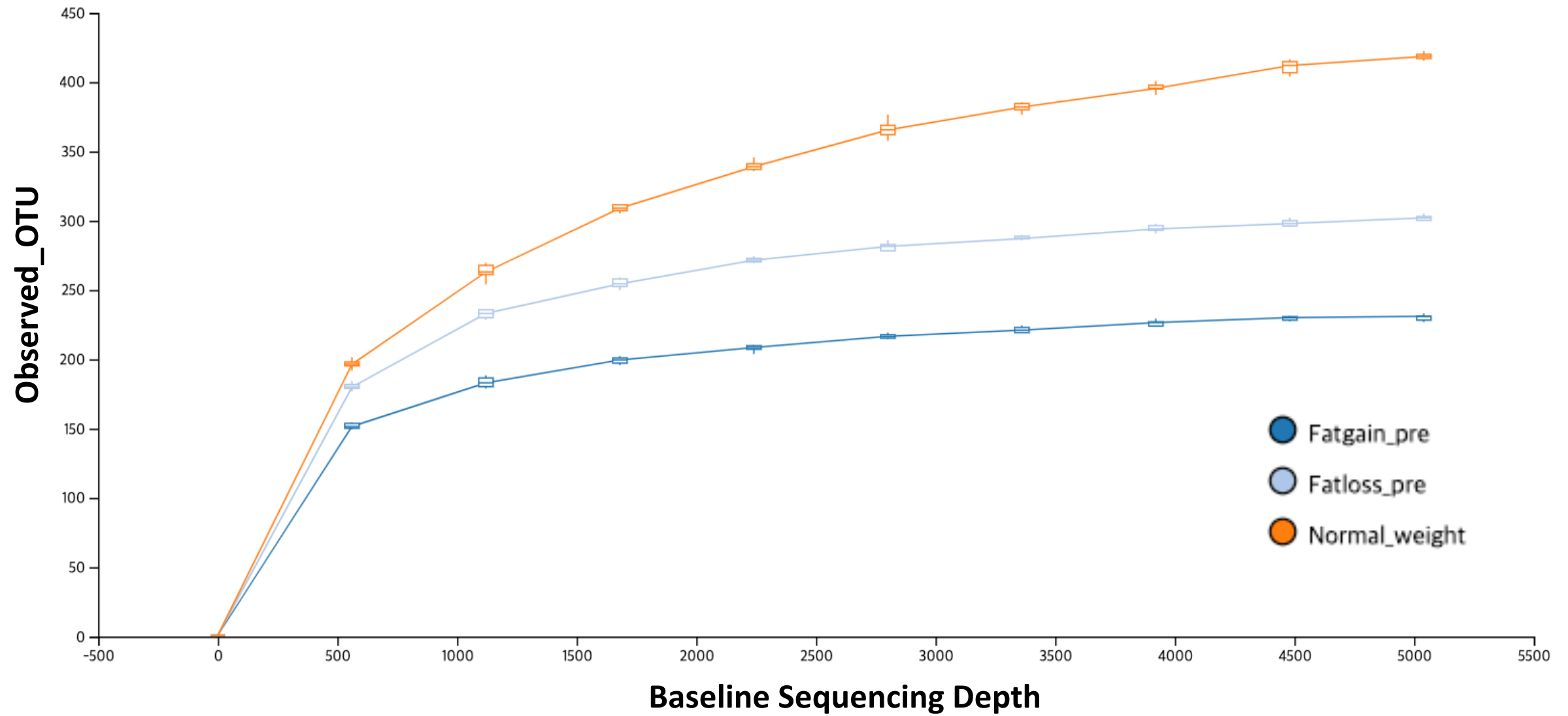

**Fig S1b**

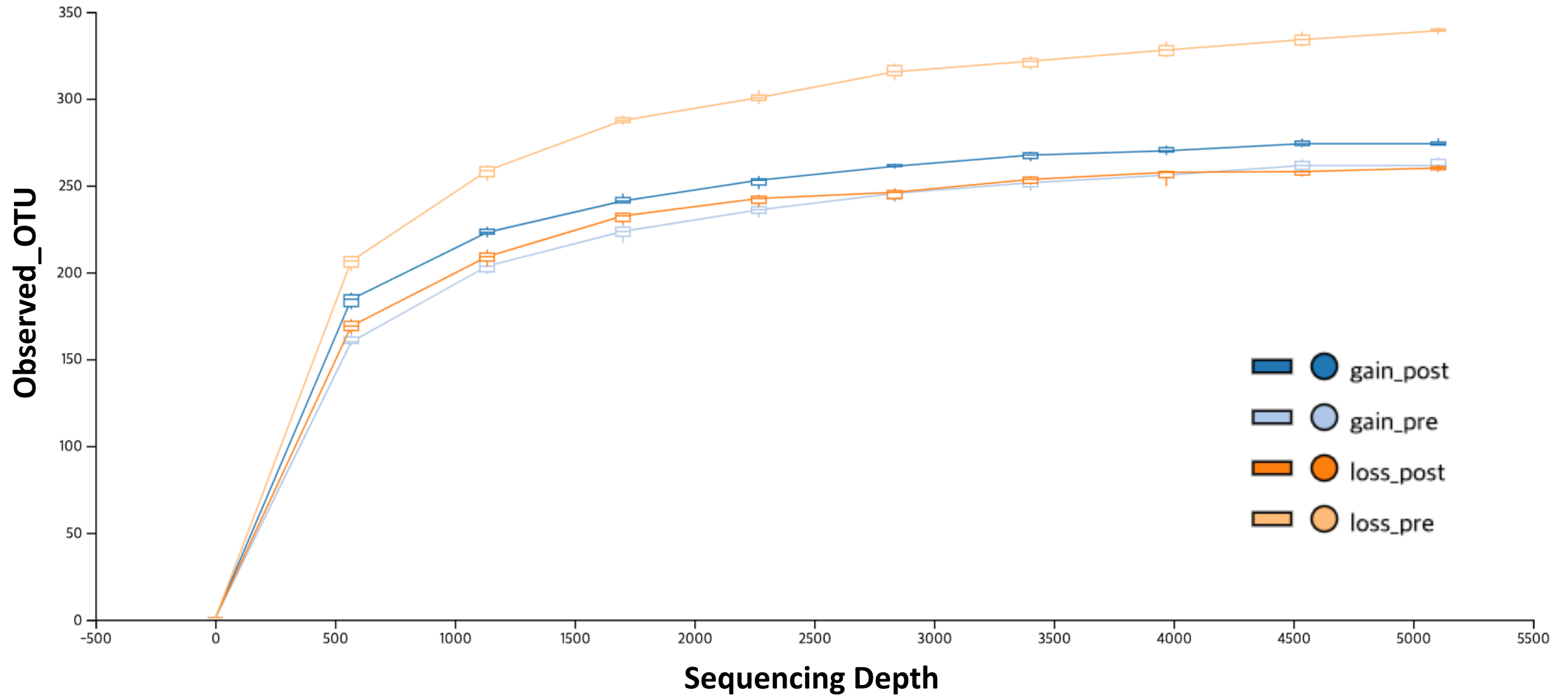

**Fig S2a**

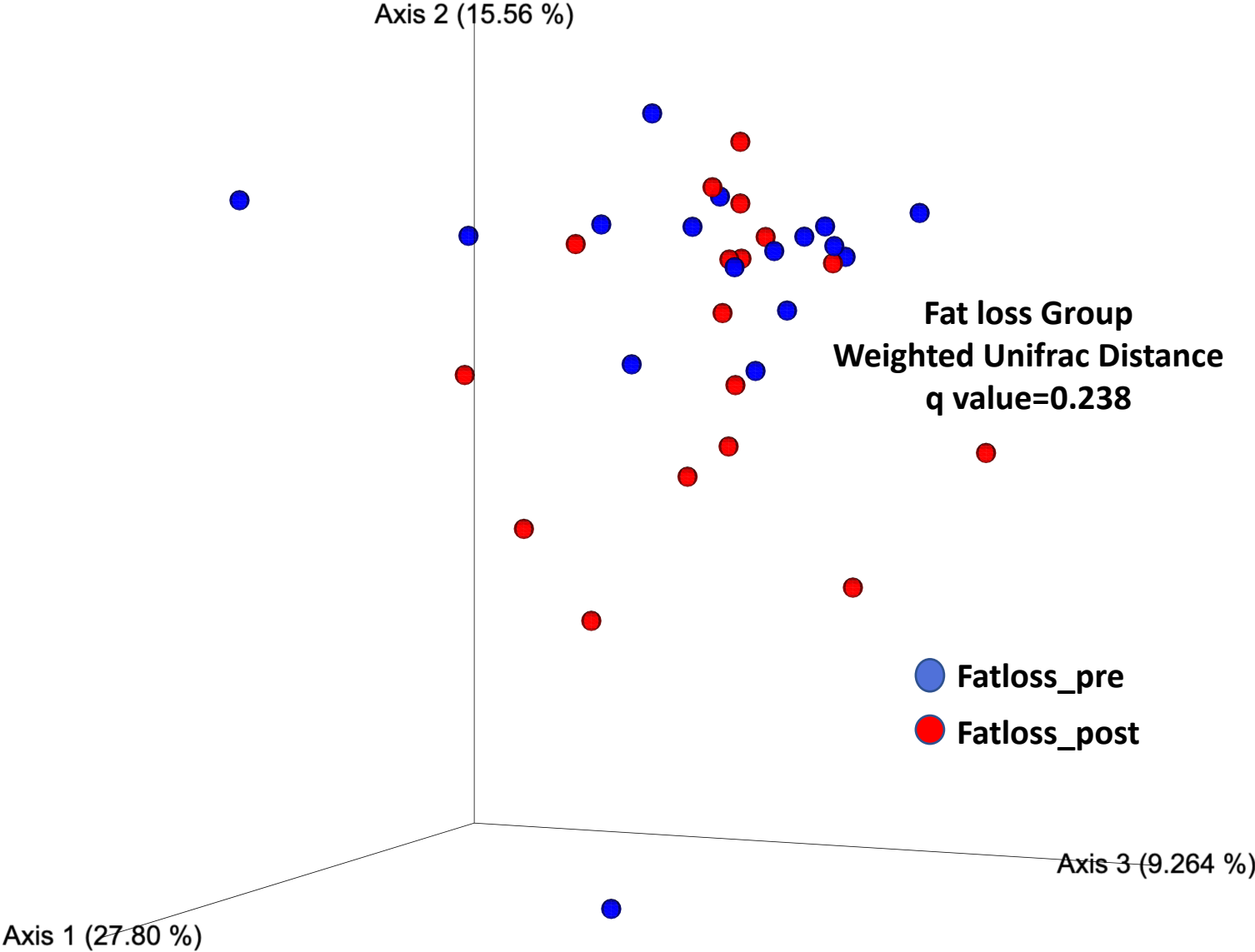

**Fig S2b**

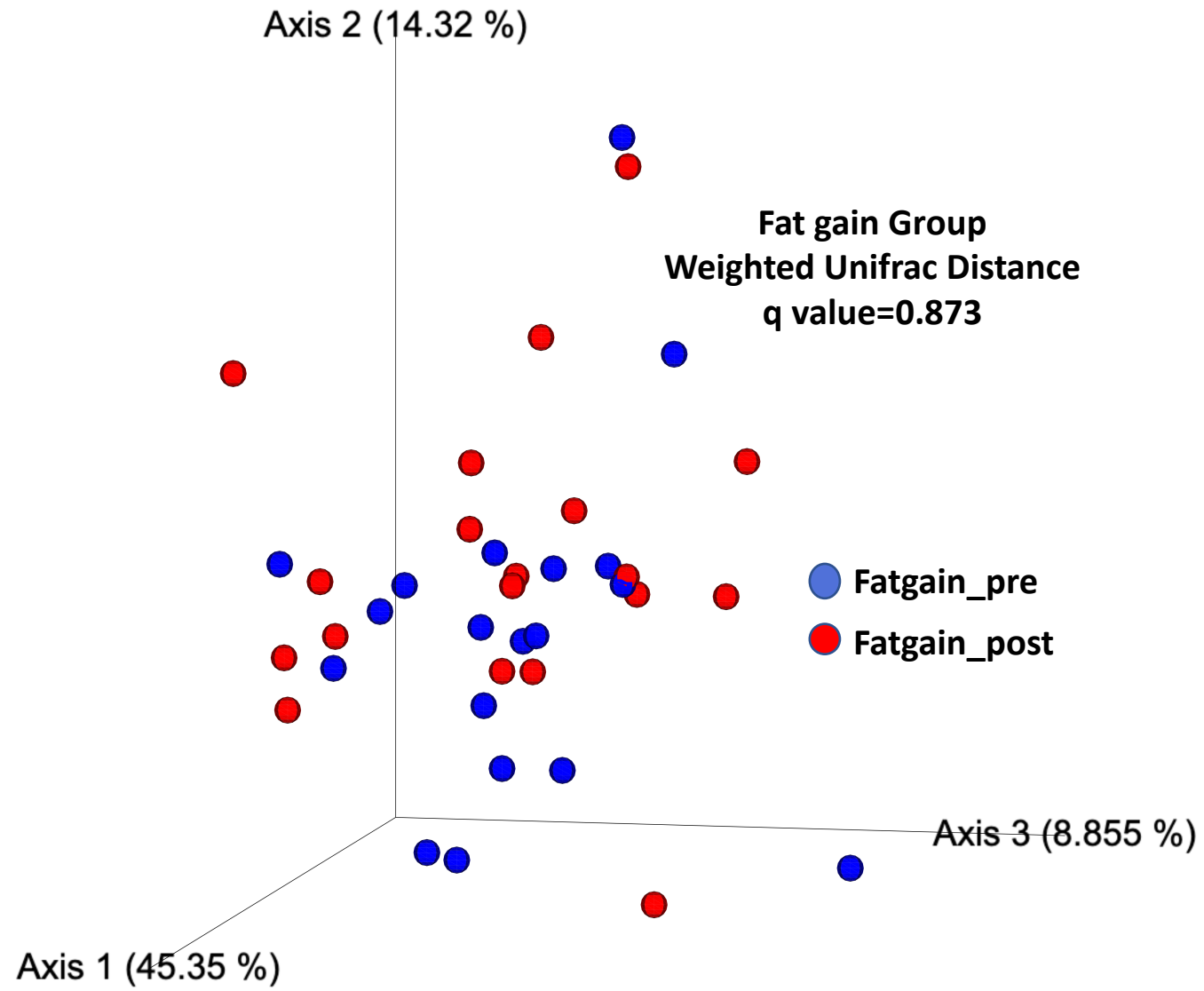

Supplement: Supplementary file 3 — Additional file 3: Fig. S1. The alpha rarefaction plots for the observed_OTUs index in the baseline study, including the controls and preintervention stages of the fat loss and fat gain groups (a), and the intervention study, including the pre- and postintervention stages in the fat loss and fat gain groups (b). Fig. S2. Principal coordinate analysis based on the weighted Unifrac distance between the pre- and postintervention stages in the fat loss (a) and fat gain groups (b). [file 12866_2020_2002_MOESM3_ESM.pdf]
